# Biological evidence of cancer stem-like cells and recurrent disease in osteosarcoma

**DOI:** 10.20517/cdr.2021.130

**Published:** 2022-02-16

**Authors:** Camille Jubelin, Javier Muñoz-Garcia, Denis Cochonneau, Emilie Moranton, Marie-Françoise Heymann, Dominique Heymann

**Affiliations:** ^1^CNRS, US2B, UMR 6286, Nantes Université, Biological Sciences and Biotechnologies unit, Nantes 44000, France.; ^2^Institut de Cancérologie de l’Ouest, “Tumor Heterogeneity and Precision Medicine” Laboratory, Saint-Herblain 44805, France.; ^3^Atlantic Bone Screen, Saint-Herblain 44800, France.; ^4^Department of Oncology and Metabolism, University of Sheffield, Medical School, Sheffield S10 2RX, UK.

**Keywords:** Cancer stem cells, bone sarcoma, soft tissue sarcoma, drug resistance, tumor microenvironment, recurrent disease, residual disease

## Abstract

Sarcomas are a large family of cancers originating in the mesenchyme. Composed of more than 100 histological subtypes, soft tissue and bone sarcomas remain clinically challenging, particularly in children and adolescents in whom sarcomas are the second most common malignant entities. Osteosarcoma is the main primary bone tumor in adolescents and young adults and is characterized by a high propensity to induce distant metastatic foci and become multi-drug resistant. The innate and acquired resistance of osteosarcoma can be explained by high histological heterogeneity and genetic/molecular diversity. In the last decade, the notion of cancer stem-like cells (CSCs) has emerged. This subset of cancer cells has been linked to drug resistance properties, recurrence of the disease, and therapeutic failure. Although CSCs remain controversial, many elements are in favor of them playing a role in the development of the drug resistance profile. The present review gives a brief overview of the most recent biological evidence of the presence of CSCs in osteosarcomas and their role in the drug resistance profile of these rare oncological entities. Their use as promising therapeutic targets is discussed.

## INTRODUCTION

Sarcomas are composed of highly heterogeneous soft tissue and bone oncological entities that are members of the mesenchymal tumor family^[1,2]^. Osteosarcoma is the main bone sarcoma, with high prevalence in adolescents and young adults. Two peaks of incidence are described in the literature, a main peak around 18 years and a second in the sixth decade of life, more frequently diagnosed in patients following Paget’s disease or radiotherapy and referred to as secondary osteosarcomas^[2-4]^. The conventional therapeutic regimen for osteosarcoma is based on a sequential approach combining surgery and neoadjuvant and adjuvant polychemotherapies^[5]^. Considered to be radioresistant, radiotherapy is nevertheless part of the therapeutic arsenal, proposed in osteosarcomas for which the surgical procedure is delicate, such as tumors in high-risk locations, and can be used for better local control of the tumor^[6]^. Unfortunately, the therapeutic response in osteosarcoma patients has not improved in the last four decades, with an overall survival rate of around 70% after five years for localized disease. This rate drops dramatically to 30% when lung metastases can be detected^[7]^.

As described in other types of cancer, osteosarcoma evolves under the pressure of random mutational changes^[8,9]^, with preferential clonal proliferation and epigenetic modifications^[10-13] ^within the clonal population, leading to genetic instability, high genetic diversity, and high tumor heterogeneity^[13,14]^. Therapeutic failure is frequently attributed to this intratumoral heterogeneity, and more specifically to the emergence of oligoclonal tumor cells capable of evading the therapeutic drugs. From this observation the concept of CSCs has emerged, in reference to embryonic stem (ES) cells. CSCs express transcription factors (e.g., Nanog, Oct4, and Sox2) initially detected in ES cells and exhibit pluripotent differentiation properties into various functional cells able to reconstitute the complete tumor mass. The tumor-initiating cells, the CSCs, have been described as tumor cells capable of reproducing all features of the initial tumor mass and have been associated with tumor recurrence, propagation, and drug resistance^[15-18]^. Unfortunately, long-term relapse in patients considered clinically disease-free is observed in numerous cancers, including osteosarcoma^[19,20]^. Minimal residual disease is defined as malignant cells that are resistant to treatment and that remain in patients after remission, leading to relapse and metastasis. Minimal residual disease is composed of drug-resistant tumor cells and is presented dynamically as persister/dormant/quiescent/cancer cells in residual tumors, such as circulating tumor cells in peripheral blood and disseminated tumor cells in bone marrow and other metastatic sites^[13-15,21]^. In this context, tumor recurrence may be related to the persistence of CSCs. Increasing evidence highlights the existence of CSCs in osteosarcomas, although their real contribution to pathogenesis remains speculative. The present review aims to give a brief overview of the most recent knowledge available on CSCs in osteosarcoma and their potential clinical interest as new therapeutic targets.

## PROPERTIES OF CANCER STEM-LIKE CELLS IN OSTEOSARCOMA AND BIOLOGICAL *IN VIVO* EVIDENCE

Around 5% of osteosarcoma patients develop local recurrence of their disease between 6 and 28 months after their first line of treatment and disease-free survival of up to 12 years is usually observed in 46% of patients^[22]^. A large series confirmed a relatively low rate of local recurrence of high-grade osteosarcoma in contrast to the relapse disease associated with lung metastases^[23,24]^. In 2010, Perrot *et al.*^[20]^ described local recurrence with metastatic foci in patients with telangiectatic osteosarcoma of the humerus after 13 years of complete remission. The local recurrence exhibited the same histological subtype as the initial tumor and was observed at the injection site of autologous fat grafts that had been performed 18 months before the recurrence for plastic surgery. More recently, Pennati *et al.*^[25]^ studied a series of autologous fat grafts in sarcomas and did not exclude an increased risk of local recurrence after the fat grafting procedure. These clinical cases raise the question of the persistence of cancer cells that remain quiescent at the primary tumor site during the remission phase and are reactivated by alteration to their local microenvironment. Interestingly, in 2018, Le Nail *et al.*^[26]^ identified osteosarcoma cells with CSC properties from high-grade osteosarcoma samples. Of the isolated cells, two showed a high ability to form spheroids, and, even though they were not tumorigenic, these cells supported tumor growth when they were co-inoculated with human osteosarcoma cell lines in immunodeficient mice.

Asymmetric cell division has been described in stable cancer cell lines, leading to the development of proliferating and quiescent cells that were functionally related to sensitive and drug resistant cells, respectively^[15]^. The identification of CSCs in osteosarcoma has been extensively described in the literature [Table 1]. CSCs express CD24^[27]^, CD177^[28-31]^, Stro-1^[28-31]^, CD133^[32-39]^, and ALDH1^[39,41-43]^ and show specific metabolic properties^[44-47]^. Telomerase (hTert) controls the lengthening of chromosome telomeres by catalyzing the addition of repetitive DNA sequence to their end. CD271 and Stro-1 were enriched in hTert and showed metabolic specificities such an uncoupling Warburg under hypoxia^[31,47]^. In addition, as expected, these cells, which expressed stemness markers (e.g., *Nanog*, *OCT4*, and *Sox2*), were able to form spheroids *in vitro* and exhibited the properties of tumor-initiating cells in preclinical mouse models^[47]^. Among the other metabolic particularities, CSCs exhibit high aerobic glycolysis and oxidative phosphorylation^[45]^, a downregulation of the citrate cycle, and increased oxidative glutathione levels^[46]^ and show more generally an upregulation of most amino acid metabolisms^[44,46]^. A drug resistant profile has been associated with the stemness properties of CSCs, which can be modulated by epigenetic mechanisms such as DNA and mRNA methylation^[48,49] ^and with an increase in ALDH activity and ABC transporter expression^[50,51]^. Interestingly, anti-cancer therapies based on cytotoxic agents result in enrichment of CSCs in cancer cells, highlighting the potentially harmful link between CSCs and the establishment of drug resistance^[52-54]^. CSCs may be a specific subset of tumor cells with high potential for tumor-initiation and self-renewal, as has been recently observed in all primary cultures from cases of patient-derived Ewing sarcoma^[55]^.

**Table 1:** Biological characteristics and functional properties of CSCs identified in human osteosarcoma

**Biomarkers studied**	**Biological properties**	**Models**	**Ref.**
CD24	- Sphere formation - Expression of stemness markers (Oct4, Nanog, Sox2, BMI1) - Properties of tumor-initiating cells - Drug resistance	- MNNG-HOS, U2OS, MG-63, and OSC228 human cell lines - Primary cultures of human cancer cells	[27]
CD117, Stro-1	- Expression of stemness markers (CD133, CXCR4, Nanog, Otc4)	- K7M2 mouse cell line	[28]
	- *In vivo* properties of tumor-initiating cells	- 318-1, P932, and K7M2 mouse cell lines and KHOS and MNNG/HOS human cell lines	[29]
	- Drug resistance (ABCG2): resistance to methotrexate, cisplatin	- U2OS human cell line	[30]
		- MG63, MNN/HOS, and 143B human cell lines and patient-derived cells	[31]
CD133	- Sphere formation - Expression of stemness markers (Sox2, Oct3/4, Nanog)	- SaOS2, MG63, and U2OS human cell lines	[32]
	- Expression of ABCG2 and MDR1	- Primary cultures of human cancer cells and MG63 human cell line	[33]
	- Expression of ABCB1, ABCC2, and the metastasis-associated genes β4-integrin, ezrin, MMP-13, and CXCR4	- FFFE samples and MG63 human cell line	[34]
	- Concomitant CD133/CXCR4 expression significantly associated with lung metastasis	- SaOS2 human cell line	[35,36]
	- Expression of CD133 and ALDH1 positively associated with lymph node metastasis and distant metastasis	- FPPE samples and SaOS2, U2OS, MG63, HOS, MNNG/HOS, HuO9, and 143B human cell lines	[37]
		- FFPE samples	[38,39]
CD271	- Sphere formation - Ability for self-renewal - Resistance to DDP therapy - Overexpression of Nanog, Oct3/4, STAT3, DNA-PKcs, Bcl-2, and ABCG2 - *In vivo* tumorigenicity	- FFPE samples and U2OS, MNNG/HOS, and SaOS2 human cell lines	[40]
ALDH1	- Sphere formation - Ability for self-renewal	- FPPE samples	[39]
	- Expression of stemness markers (CD133, CXCR4, Nanog, Otc4, Sox2, KLF4)	- MG63 human cell line	[41]
	- Drug resistance	- HuO9, OS99-1, MG63, and SaOs2 human cell lines	[42]
	- *In vivo* tumorigenicity	- HOS, MG63, MHM, MNNG/HOS, OHS, and U2OS human cell lines	[43]
hTERT enrichment	- Expression of CD117 and Stro-1 - Spheroid formation	- Primary osteosarcoma cell lines (OS1-4) - MG63, MNNG/HOS, and 143B human cell lines	[31]
Metabolic properties	- Specific metabolic feature of osteosarcoma stem-like cells: amino acid, fatty acid, energy, and nucleic acid	- 143B and MG63 human cell lines	[44]
	- Involvement of the Rap1 and Ras signaling pathways in methotrexate resistance	- OS13 human cell line	[45]
	- High aerobic glycolysis and oxidative phosphorylation: association to LINB28 expression	- HOS human cell line	[46]
	- Downregulation of the citrate cycle and elevation of oxidized glutathione levels - Upregulation of most of the amino acid metabolisms - Uncoupling Warburg and stemness in CD133^+^ cells under hypoxia	- SaoS2 human cell line	[47]
N-methyltransferase	- Sphere formation - Expression of CD133, CD44, Oct4, Sox2, Nanog, Nestin, ABCG2, and BMI-1	- MG-63 human cell line	[48]
m^6^A methylome	- Multidrug resistance - Sphere formation - Overexpression of CD117, stro-1, CD113, and stemness markers (*SOX2*, *POU5F1*, *NANOG*, *KLF4*) - Upregulation of *METTL3* and *ALKBH5* and downregulation of *METTL14* and *FTO*	- MG63 human cell line	[49]

## MOLECULAR REGULATION OF CANCER STEM-LIKE CELLS IN OSTEOSARCOMA

**Figure 1:**
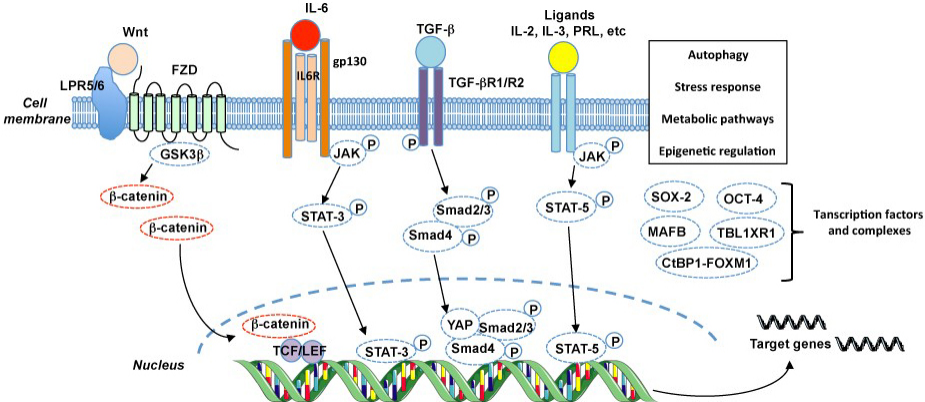
Main signaling pathways and mechanisms regulating the maintenance of cancer stem-like cells in osteosarcoma. LPR: Lipoprotein receptor-related protein; FZD: frizzled receptor; PRL: prolactin receptor.

Osteosarcoma growth and the distant dissemination of cancer cells are controlled by a permanent dialog between cancer cells and their microenvironment^[2,56]^. These soluble and membranous mediators trigger specific intracellular molecular cascades that lead to control of cellular processes, including cell death, epithelial-mesenchymal transition, or spreading, but also proliferation and quiescence. In this context, the behavior of CSCs is controlled by the tumor microenvironment. In recent decades, key signaling pathways regulating CSCs have been identified and become the source of therapeutic development [Figure 1].

The **Wnt/β-catenin** pathway contributes to the regulation of numerous cellular processes (e.g., proliferation, differentiation, and polarization) and is thus strongly associated with embryonic development. The Wnt glycoprotein family is composed of 19 secreted members that interact with cell membranes after binding to 1 of the 10 Frizzled receptors identified which are G protein-coupled receptors or to a co-receptor such as LRP-5 or -6 or tyrosine kinase receptor chains including retinoic acid-related orphan receptor and RyK. In the absence of Wnt ligand, β-catenin is degraded by the proteasome after sequestration associated GSK-3β, and the Wnt/β-catenin pathway is considered as inactive. The Wnt/β-catenin pathway is activated by the binding of one Wnt ligand to its receptor/co-receptor complex that leads to a series of phosphorylation cascades and recruitment of the receptor chains and then to the inactivation of the β-catenin degradation process. Consequently, β-catenin accumulates to the cytoplasm and is translocated into the nucleus before interacting with transcription factors, members of the TCF/LEF family, and activating target genes [Figure 1]. Any disturbance (e.g., mutations or activation) in this molecular pathway leads to pathological situations^[57]^. Recently, Deng *et al.*^[58]^ studied the involvement of Indian hedgehog (IHH) signaling in cartilage and bone tumors by deleting *Ptch1* encoding an inhibitor of IHH receptor. They demonstrated that deleting *Ptch1 *in mice was associated with an increase in Wnt member expression and the development of skeletal diseases, including osteosarcoma. Interestingly, inhibiting the Wnt/β-catenin pathway abolished the development of osteosarcoma, highlighting the key role played by this molecular pathway in the pathogenesis of bone sarcomas^[58]^. The Wnt/β-catenin pathway might be the link among tumor development, drug resistance, and CSCs in osteosarcoma. Whether or not the Wnt/β-catenin cascade was related to chemoresistance, it appeared to be a driver of cancer by acting directly on tumor cells, but also by modulating the immune microenvironment^[59]^. This cancer driver is persistently activated in the CSCs of osteosarcoma, and the stemness properties induced by chemotherapies are related to activation of the Wnt/β-catenin cascade^[43,60,61]^. In this context, most molecular machineries that modulate the expression level of Wnt/β-catenin may affect cancer cell behavior. Thus, epigenetic regulation of Wnt/β-catenin using the lncRNA DLX6-AS1/miR-129-5p/DLK1 axis or histone methyltransferase SETD2 results in increased stemness properties for osteosarcoma cells, tumor growth, and drug resistance^[62,63]^. The key contribution of Wnt/β-catenin in the maintenance of CSCs may lead to the development of new targeted therapies in osteosarcoma, as described below.

**IL-6/STAT3 **signaling has also been identified as a crucial regulator of bone remodeling and primary bone tumors^[64]^. The IL-6 family of cytokines, composed of 10 members including IL-11, OSM, and LIF, induces redundant and pleiotropic activities such as embryogenesis, differentiation, or inflammation. Most of the members of this family share the transducing receptor β-subunit gp130 as part of a multimeric receptor complex that includes a specific receptor α-subunit (e.g., IL-6R). The oligomerization of receptor subunits induced by each ligand results in various transductions of signaling pathways dominated by JAK/STAT3 activation and others such as MAPKs, p38, and JNK [Figure 1]. In addition to its functions on the tumor microenvironment (e.g., bone and immune cells), the IL-6 signaling pathway controls the maintenance of CSCs in osteosarcoma^[65]^. IL-6 released by activated mesenchymal stem cells (MSCs) in the tumor microenvironment promoted osteosarcoma stemness and the spreading properties of cancer cells^[65]^. In addition, MSCs supported drug resistance through STAT-3 signaling in cancer cells activated by IL-6^[66]^. MSCs and osteosarcoma cells then established a reciprocal dialog initiated by TGF-β containing extracellular vesicles secreted by cancer cells that induced the production of IL-6 by MSCs, which in turn supported stemness, drug resistance, and tumor progression^[67]^. The use of active drugs confirmed the contribution of the IL-6/STAT3 axis in osteosarcoma stemness^[68,69]^.

**The TGF-β/Smad axis** regulates the self-renewal of osteosarcoma cells. TGF-β belongs to a large family of at least 30 secreted proteins sharing structural similarities. TGF-β growth factors are secreted as latent precursors which can bind to specific receptor chains after activation in mature form. TGF-β induces the assembly of type I and II TGF-β receptors, leading to the formation of heteromeric receptors and the initiation of the signal transduction. The type I TGF-β receptor shows intrinsic tyrosine kinase activity, phosphorylates the type II chain, and initiates the downstream signaling, which includes Smads phosphorylation. Phospho-Smads complexes are translocated into the nucleus where they cooperate with YAP/TAZ transcription regulators and modulate the transcription of target genes [Figure 1]. Zhang *et al.*^[70]^ studied the functional impact of TGF-β1 on osteosarcoma stemness in a hypoxic environment. They demonstrated the crucial role played by TGF-β1 on the proliferative state of cancer cells, which acquired the stem cell phenotype for self-renewal, drug resistance, neoangiogeneiss, and tumorigenicity; on the contrary, blocking the TGF-β1 signaling pathway reduced the dedifferentiation program of osteosarcoma cells. Similarly, by using gamabufotalin, a bufadienolide extracted from toad venom, it has recently been demonstrated that blockading the TGF-β/periostin/PI3K/AKT axis resulted in suppression of CSCs in osteosarcoma^[71]^. CSCs associated with TGF-β activity were also linked to drug resistance, as shown for EGFR inhibitors, highlighting once again the role played by CSCs in the drug resistance process^[72]^.

Recently, **a series of transcription factors **were identified as regulators of cancer stemness in osteosarcoma. The transcription factor Sox determining the region Y-box 2 **(**Sox2) plays a key role in developing and controlling the embryonic stem cell state and was identified as a biomarker for CSCs in osteosarcoma [Table 1]. In addition, the proliferation of osteosarcoma cells and tumor development requires Sox2^[73]^. Maurizi *et al*.^[73] ^compared tumor growth in Cre-bearing mice with identical Rb and p53 genotypes in a background of Sox2-deficient or wild-type mice. Tumor development was significantly slowed down in the Sox2-deficient mice compared to the other groups, and the survival rate was also higher in the Sox2 knockout mice. Sox2 appeared essential for the survival and proliferation of all osteosarcoma cells, including CSCs. The Hippo pathway, which is under the transcriptional control of Sox2, was directly related to the same activities, and deactivating Sox2 effectors (e.g., YAP) resulted similarly in a reduction in tumor growth^[73]^. Chen *et al.*^[74]^ demonstrated that musculoaponeurotic fibrosarcoma oncogene homolog B (MAFB) is highly expressed in osteosarcoma and more specifically in CSCs, and this transcription factor, similar to Sox2, is required for the proliferation and tumorigenicity of osteosarcoma cells. Interestingly, they observed that maintaining the self-renewal potential of CSCs was under the transcriptional control of Sox-9, a stem cell regulator^[74]^. More recently, STAT-5 associated signaling was identified as a key regulator^[75]^. The knockdown of STAT-5 (A and B isoforms) using an siRNA approach reduced pimozide-induced tumor growth in mice, in addition to suppressing *in vitro *sphere formation. Inhibiting STAT-5 signaling thus impaired osteosarcoma self-renewal and development^[75]^. JAK/STAT-5 activation belongs to the downstream signaling associated with various cytokine/hormone-induced signaling pathways, including prolactin, IL-2, IL-3, *etc*. Oct4 promoted osteosarcoma development by supporting the maintenance of CSCs through the increase in AK055347, a long-noncoding (lnc) RNA. Oct4 knockdown with siRNA induced a significant decrease in cell proliferation, invasion, and apoptosis^[76]^. TBL1XR1 is a transcriptional co-factor which is overexpressed in osteosarcoma patients^[77]^. Its overexpression in MG63 and U2-OS cell lines induced a CSC phenotype in contrast to its repression. TBL1XR1 thus provides osteosarcoma cells with tumorigenic properties and promotes the recurrence of osteosarcoma in a STAT-3 signaling dependent manner^[77]^. Transcriptional complexes can also modulate osteosarcoma drug resistance. Thus, the CtBP1-FOXM1 transcriptional complex increased *MDR1* expression in osteosarcoma CSCs, which is associated with drug resistance^[78]^. Interestingly, small molecules targeting this complex reversed the MDR1-mediated resistance both *in vitro* and in murine preclinical models.

Regulating osteosarcoma growth through the oct4/lncRNA axis highlights the epigenetic regulation of osteosarcoma CSCs^[79]^. This observation is supported by the rich literature emerging in the last 10 years^[76] ^[Table 1]. In this context, chromodomain helicase DNA binding protein 1-like significantly reduced osteosarcoma proliferation and drug resistance though its binding to DNA. It also controls chromosomal integrity maintenance, DNA repair, and transcriptional regulation^[79]^. Ubiquitin-specific peptidase 39 is a crucial factor for assembling mature spliceosome complex, and its knockdown leads to the inhibition of osteosarcoma cell proliferation combined with an increase in apoptosis^[80]^. Human antigen R is involved in stabilizing mRNA, and its repression in osteosarcoma cells reduced their stemness properties and increased the drug response^[81]^. These activities were related to YAP activation. Several recent publications showed the role played by specific miRNA in controlling stemness in osteosarcoma, including miR29b and its target Spin1^[82]^, miR34a^[83] ^and the DNMT1/miR34a/Bcl2 axis^[84,85]^, TNF-α-miR155 signaling^[86]^, miR335 and its target POUF5^[87]^, miR429 and its target Sox2^[88]^, and the TGF-β/miR499a/SHKBP1 89 axis^[89,90]^. Very recently, leukemia inhibitory factor (LIF) was shown to belong to the IL-6 family of cytokines, similarly activating STAT-3, and was recently revealed as a super-enhancer-controlled regulator of CSC properties, confirming the role of STAT-3 transcription factor in the functional regulation of CSCs in osteosarcoma^[91]^. TSSC3 tumor-suppressing STF cDNA 3 (TSSC3), the first apoptosis-related gene reported to be imprinted, repressed the self-renewal of osteosarcoma CSCs^[92]^. Finally, lncRNAs also play a part in the biological regulation of CSCs in osteosarcoma^[76,92,93]^.

**Autophagy** ^[94,95]^, **stress response**^[96-98]^, and **numerous enzymatic pathways**^[99-104] ^complete the landscape of the osteosarcoma CSC regulation mode. Autophagy was shown as a critical biological process for maintaining CSCs in OS^[94]^, and defective autophagy was directly associated with the decrease in CSCs^[95]^. Similarly, the knockdown of stress-induced phosphoprotein 1 resulted in the inhibition of CSC invasiveness and migration^[96]^. STIP-1 is a co-chaperone that binds to HSP70 and -90 and consequently inhibits Hsp90 by 17-AAG-reduced stem cell-like properties and decreased drug resistance in OS^[97]^.

## THERAPEUTIC TARGETING OF CANCER STEM-LIKE CELLS IN OSTEOSARCOMA

The recent evidence of CSCs in osteosarcoma and better understanding of the molecular pathways required for their maintenance, led to the identification of new therapeutic targets, as summarized in Table 2.

**Table 2:** Potential therapeutic approach to CSCs in osteosarcoma

**Drug**	**Molecular pathway involved or therapeutic approaches**	**Ref.**
**Wnt/β-catenin targeting**		
Tankyrase inhibitor (IWR-1)	Attenuation of Wnt/β-catenin signaling	[105]
Tegavivint	β-catenin/transducing β-like protein 1 (TBL1) inhibition	[106]
Dioscein	Akt/GSK3/β-catenin	[107]
Tideglusib	GSK-3β/NOTCH1	[108]
**TGF-β/BMP2 targeting**		
Gamabufotalin	TGF-β/periostin/PI3K/AKT	[109]
BMP2	BMP2 receptor signaling	[110]
**Other receptor signaling targeting (STAT-3, STAT-5, ER-α, TRAF-2, *etc.*) and transcription factors**		
Bruceine D	STAT-3 inhibition	[111]
Pimozide	STAT-5 signaling	[75,112]
Decitabine	Activation of estrogen receptor alpha (ER-α)	[113]
NCB-0846	TRAF2- and NCK-interacting protein kinase	[114]
Melatonin	Suppression of SOX9 mediated signaling	[115]
Statins	KLF4	[116]
**Targeting of kinase activities**		
Fasudil	Rho-associated coiled-coil containing kinase (ROCK) inhibition	[100]
**Autophagy and metabolic targeting**		
Thioridazine	Autophagy	[94]
Metfomin	- Inhibition of mitochondrial functions (decrease in oxygen assumption, decreased mitochondrial membrane potential, decreased ATP production)	[117]
	- Pyruvate kinase isoenzyme M2 (PKM2)	[118]
	- ROS-mediated apoptosis and autophagy	[119]
	- Activation and phosphorylation of the energetic sensor AMPK	[120]
Wogonin	ROS regulation	[121]
DMAMCL	Cell cycle	[122]
DAPT	γ-secretase inhibition	[123]
**Combinations with chemotherapy and sensitization to chemotherapy**		
Ascorbate	Sensitization to cisplatin	[124]
Ouabain	Sensitization to cisplatin: Na^+^/K^+^ ATPase inhibition	[125]
Tangeretin-assisted platinum nanoparticles	Combination with doxorubucin	[126]
Senolytic drug (Fisetin)	Combination with etoposide	[127]
**Immunotherapy**		
Immunotherapy based on cytokine induced killer cells	CSCs spared after chemotherapy or other targeted therapies	[128,129]
**Modulation of epigenetic events**		
Epigenetic targeting	- USP39 silencing	[80]
	- HuR knockdown	[81]
	- Disruption of the DNMT1/miR34a/Bcl-2 axis by isovitexin	[85]
	- lncRNA HOXD-AS1 knockdown	[92]
	- RAB39A silencing	[99]
	- Targeting of lncRNA SOX2OT variant 7 by EGCG (polyphenol isolated from green tea)	[130]
**Photo therapy**		
- Graphene oxide nanoparticle-loaded ginsenoside Rg3	Photodynamic therapy	[131]
- CD271 antibody-functionalized HGNs	Photothermal therapy	[132]
**Drug delivery systems**		
- Salinomycin-loaded PLA nanoparticles	Delivery of solinomycin	[133]
- Lipid-polymer nanoparticles with CD133 aptamers	Delivery of all-trans retinoic acid	[134]
- Lipid-polymer nanoparticles with EGFR and CD133 aptamers	Delivery of salinomycin	[135]

Repressing the signaling pathways related to the maintenance of CSCs (see Table 1) resulted in the slowdown of tumor growth and inhibition of the metastatic process^[105-116]^. As previously mentioned, Wnt/β-catenin appeared crucial for the maintenance of CSCs and its attenuation by using tankyrase inhibitor, or tegavivint was associated with a decrease in both CSC numbers and tumor progression^[105,106]^. GSK3 appeared highly expressed in osteosarcoma and targeting Akt/GSK3/β-catenin or Akt/GSK3-/Notch-1, respectively, with dioscein or tideglusib repressed CSC and tumor growth^[107,108]^. Gamabufotalin-induced similar activities by targeting TGF-β/periostin/PI3K/Akt signaling as it has been shown for hepatocarcinoma^[71,109]^. Similar results were obtained by targeting BMP2R^[110]^. Drugs targeting transcription factors (e.g., STAT-3 and STAT5) controlling the development of CSCs may also be used to improve the therapeutic approaches to osteosarcoma^[75,111,112]^. Activation of hormone signaling can reduce stemness in osteosarcoma, as shown by the activation of estrogen receptor alpha by decitabine^[113]^. Most cytokine-induced signaling pathways result in the translocation of transcription factors which modulate the transcription of target genes. Targeting of such transcription factors (e.g., KLF4 and Sox9) may be used for reducing CSCs in osteosarcoma^[114-116]^. Similarly, ROCK inhibition by fasudil suppressed* in vitro* cell proliferation and reduced their tumorigeneicity *in vivo*^[100]^. Cell metabolism is significantly modulated in CSCs (e.g., autophagy and cell cycle), and these specificities can be used for targeting CSCs in osteosarcoma. For instance, thioridazin and metformin target autophagy and metformin and wogomin modulated ROS-mediated apoptosis in CSCs and resensitize CSCs to cell death^[114-116]^. Similarly, regulation of cell cycle by DMAMCL or inhibition of γ-secretase by DAPT affects the behavior of CSCs and their function in tumor growth^[122,123]^.

Drugs/effective agents can be used as sensitization agents to chemotherapy^[124,125]^ or in combination with chemotherapeutic drugs^[126,127]^. Numerous cytokines are involved in the control of local immunity of cancer cells^[128]^ and immunotherapies have been proposed for targeting CTCs^[129]^. Specific silencing of the epigenetic partners of CSCs can induce similar regression in tumor growth and metastatic development by altering CSC maintenance^[80,81,92,95,99,129]^. Nanoparticles can be used for developing phototherapies and drug delivery systems. In this context, nanoparticles have been functionalized and adapted for phototherapy with a specific aim to improve the targeting of CSCs using^[131,132]^. Finally, drug delivery systems have also been proposed^[133-135]^. For all these therapeutic approaches, the question of the general toxicity in healthy tissue stem cells and the specificity of the targeting remains unanswered.

## CONCLUSION

Long considered as controversial, today CSCs are a realistic therapeutic target in osteosarcoma^[1,2]^. Osteosarcoma remains a highly heterogeneous oncological entity in perpetual evolution due to a strong clonal dynamic^[136]^, leading to very efficient adaptation to drugs and the establishment of drug resistance^[15]^. The dynamic properties of tumor evolution have led to numerous questions about CSCs and their functional impact: (1) Can we detect CSCs in the bloodstream and can we use circulating tumor cells to follow the minimal residual disease and identify personalized therapeutic options^[137]^? (2) Are CSCs capable of migrating to distant organs to establish metastatic foci? (3) Is the dynamic evolution of osteosarcoma similar in the primary site and in the metastatic foci? (4) What is the functional regulation of CSCs and are they under the control of proliferating osteosarcoma cells? (5) Are CSCs regulated by the tumor microenvironment and by which molecular pathways? (6) Can we use immune therapies in combination with other drugs (e.g., chemotherapy) to target CSCs and improve overall survival in osteosarcoma? (7) How can we specifically control CSC metabolism and consequently can we set up specific therapeutic options to control CSC wake-up? (8) As osteosarcoma is a form of cancer that originates in the mesenchyme, can we use the fibrogenic reprogramming of CSCs as a therapeutic option^[138]^? Even whether sarcomas being considered as an immune desert explaining the current poor clinical efficacy of immune therapies needs more research^[1,128]^, macrophage and stromal cells contribute to the establishment of drug resistance and may be identified as therapeutic target in osteosarcoma^[139]^. For instance, M2 macrophage may be associated with tumor angiogenesis. Tumor cells release a high number of protons that induce local acidosis, favoring the release of inflammatory mediators by local stromal cells, which in turn facilitates tumor invasiveness and metastasis in osteosarcoma^[140]^. Overall, it has been demonstrated that stromal cells significantly contribute to increase the stemness properties of osteosarcoma cells by inducing metabolic reprogramming of cancer cells^[141,142]^. Consequently, stromal cells constitute an interesting reservoir of stemness targeting to reduce osteosarcoma progression, as has been shown recently^[143]^. A better understanding of the role of stromal cells in the control of stemness would help to identify new mediators associated with stemness, drug resistance, and tumor progression. Overall, CSCs are promising targets in osteosarcoma, as demonstrated by the most recent data described in this review, paving the way for a new therapeutic era focused on better-controlled residual disease in osteosarcoma through targeting CSCs.

## DECLARATIONS

### Copyright

© The Author(s) 2022.
